# RNA-mediated inhibition of mitochondrial SHMT2 impairs cancer cell proliferation

**DOI:** 10.1038/s41420-025-02646-y

**Published:** 2025-08-06

**Authors:** Francesca Romana Liberati, Sharon Spizzichino, Sara Di Russo, Giulia Elizabeth Borsatti, Agnese Riva, Maria Chiara Magnifico, Amani Bouzidi, Giorgio Giardina, Marzia Arese, Chiara Scribani Rossi, Dalila Boi, Giovanna Boumis, Federica Di Fonzo, Giulia Guarguaglini, Roberto Contestabile, Angela Tramonti, Alberto Macone, Alessandro Paiardini, Serena Rinaldo, Alessio Paone, Francesca Cutruzzolà

**Affiliations:** 1 Department of Biochemical Sciences A. Rossi Fanelli, Sapienza University of Rome, Rome, Italy; 2 Translational Oncology Research Unit IRCCS Regina Elena National Cancer Institute, Rome, Italy; 3 Istituto di Biologia e Patologia Molecolari, Consiglio Nazionale delle Ricerche, c/o Sapienza University of Rome, Rome, Italy; 4 Laboratory affiliated to Istituto Pasteur Italia-Fondazione Cenci Bolognetti, Rome, Italy

**Keywords:** Cancer metabolism, RNA

## Abstract

Targeting metabolic reprogramming is crucial for cancer treatment. Recent advances highlight RNA’s ability to directly regulate enzyme activity through riboregulation. In this study, we used an RNA-based approach to inhibit the mitochondrial enzyme Serine hydroxymethyltransferase 2 (SHMT2), which lacks a selective in vivo inhibitor. SHMT2, often overexpressed in various cancers, is pivotal in one-carbon metabolism, a pathway vital for cell proliferation. Our results show that RNA effectively inhibits SHMT2’s serine-to-glycine conversion in vitro (IC_50_ = 4.4 ± 0.2 nM). By using a mitochondrial import signal, we successfully delivered the inhibitory RNA into the mitochondria of lung cancer cells, reducing cell viability in vitro and tumor growth in vivo in a xenograft mouse model. These findings suggest that RNA-based strategies could be extended to selectively target other RNA-binding metabolic enzymes, offering potential solutions where small molecule inhibitors fall short or to counteract drug resistance.

## Introduction

Lung cancer remains one of the most devastating malignancies worldwide, responsible for approximately 2 million new cases and 1.76 million deaths annually [1, 2]. This alarming burden underscores the need for innovative therapeutic strategies. Central to the progression of lung cancer, as well as of other tumors, is metabolic reprogramming, a hallmark of malignancy that enables tumor cells to adapt to their heightened energy and biosynthetic demands [3, 4].

Among the critical pathways supporting this metabolic adaptation is one-carbon metabolism (OCM), a network integrating serine, folate and methionine cycles [5]. OCM produces the one-carbon units required for nucleotide synthesis, methylation reactions, and redox balance processes that are essential for the rapid proliferation of cancer cells. The mitochondrial enzyme Serine hydroxymethyltransferase 2 (SHMT2) has emerged as a key player in OCM, where it catalyzes the conversion of serine to glycine, a reaction central to the production of one-carbon units [6, 7]. Although cytosolic (SHMT1) and mitochondrial (SHMT2) isoforms of SHMT catalyze an equivalent biochemical reaction, they support different functions in tumor cells. Mitochondrial serine catabolism is the principal source of one-carbon units and glycine for de novo purine synthesis and is a major source of NADPH. Moreover, SHMT2 also regulates the translation of mitochondrial respiration complexes subunits [8, 9].

SHMT2 is frequently overexpressed in various malignancies, including lung cancer, where its upregulation correlates with poor prognosis and increased tumor aggressiveness [10–14]. While small molecule inhibitors targeting SHMT2 have shown promise in preclinical studies, their clinical application has been limited by challenges such as poor selectivity, rapid clearance, and systemic toxicity [15–17]. These limitations underscore the urgent need for novel therapeutic approaches to target mitochondrial SHMT2.

RNA-based therapeutics have gained significant attention as a versatile and precise strategy for targeting cellular processes [18–20]. However, this approach has primarily focused on modulating cytoplasmic or nuclear pathways, leaving mitochondrial RNA targeting largely unexplored. The double-membrane system of the mitochondria poses a unique challenge for RNA delivery, particularly for enzymes like SHMT2 that localize and function within this organelle. A strategy that successfully targets mitochondrial enzymes could open unprecedented avenues in therapy.

Our previous work provided critical insights into a RNA-based regulatory mechanism (riboregulation) for SHMT enzymes. We demonstrated that the activity of SHMT1, the cytosolic isoform of SHMT, is regulated by the 5′ untranslated region (UTR2) of the SHMT2 mRNA through a process termed riboregulation [21]. The UTR2 RNA directly binds to SHMT1, inhibiting its serine cleavage activity while sparing the reverse glycine-to-serine reaction [21, 22]. This mechanism fine-tunes the distribution of serine and glycine across cellular compartments, supporting the metabolic adaptations required to sustain cell growth [23]. Our discovery established a new paradigm, revealing the potential of RNA molecules to riboregulate the activity of a metabolic enzyme in a highly specific manner.

In the present work we explored the therapeutic potential of this natural regulatory mechanism to target SHMT2 in cancer cells. Firstly, we have demonstrated that RNA can inhibit the SHMT2-catalyzed serine to glycine conversion in vitro. A major challenge was then to effectively deliver the regulatory RNA into mitochondria, where SHMT2 primarily functions. To address this, we successfully designed an innovative RNA-based therapeutic approach, by incorporating a mitochondrial RNA import signal [24] able to selectively transport the UTR2 RNA into mitochondria, thereby targeting SHMT2 activity within its primary site of action. This modified RNA, referred to as mUTR2, selectively inhibits SHMT2 activity in mitochondria, resulting in reduced cancer cell viability. This approach was validated in an in vivo xenograft mouse model, where mUTR2 expression leads to a significant reduction in tumor growth. This work represents two major advances in the field of nucleic acid therapeutics. It provides the first example of successful therapeutic delivery of RNA into mitochondria, overcoming a long-standing challenge in the field. Additionally, it demonstrates that RNA inhibitors can be designed to target metabolic enzymes that function as RNA-binding proteins, thus expanding the potential of RNA-based therapies beyond conventional nucleic acid-nucleic acid interactions. Altogether, these results can potentially transform the landscape of RNA therapeutics, addressing limitations of current approaches, and offering new hope for patients with hard-to-treat malignancies.

## Results

### SHMT2 is upregulated in lung adenocarcinoma

**Fig. 1:**
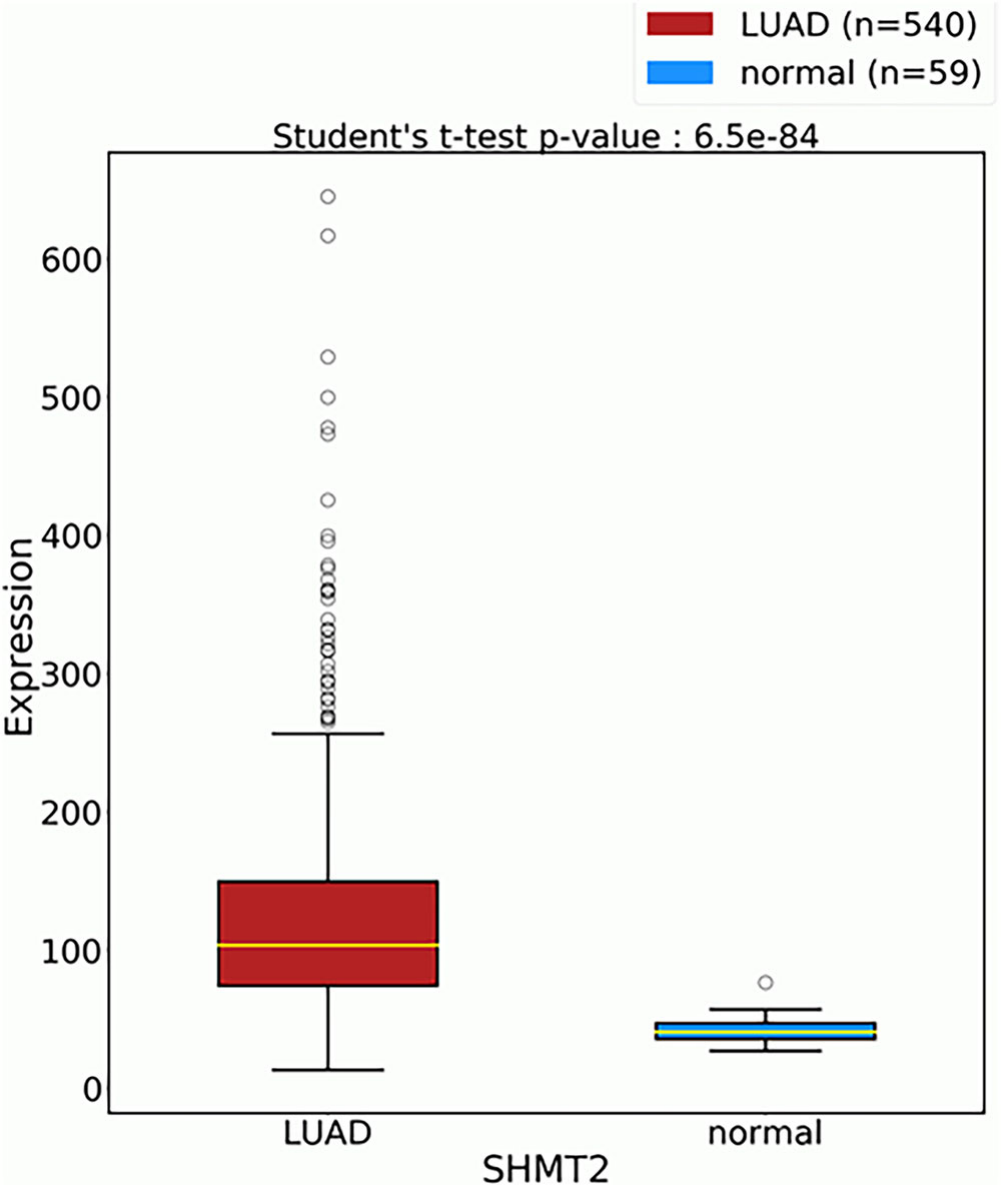
Comparison of SHMT2 expression in lung adenocarcinoma (LUAD) versus normal lung tissues. Boxplots display SHMT2 mRNA expression levels measured in TCGA samples: LUAD (*n* = 540, red) and normal (*n* = 59, blue). The yellow line within each box indicates the median; box limits represent the 25th and 75th percentiles; whiskers extend to 1.5× the interquartile range; open circles denote outliers. A two-tailed Student’s t-test indicates a highly significant increase in SHMT2 expression in LUAD compared to normal tissues (*p* = 6.5 × 10^−84^).

Initially, we aimed to assess the relevance of SHMT2 as a potential therapeutic target in lung cancer by evaluating its expression levels in healthy individuals and patients diagnosed with lung adenocarcinoma (LUAD). Our analysis retrieved from The Cancer Genome Atlas (TCGA) database revealed that SHMT2 expression was significantly elevated in LUAD tissues compared to normal lung tissues. Data in Fig. 1 clearly demonstrated a separation between the LUAD group and the normal one, with several high outliers observed in the LUAD samples. This difference reached an exceptionally high level of statistical significance, with a two-tailed p-value of 6.5e-84 (average expression in cancer samples: 127.2; in normal samples: 41.7; log2 fold change: 1.34). These findings indicate that SHMT2 plays a role in LUAD tumorigenesis, confirming its potential as a biomarker for lung adenocarcinoma, as previously suggested [25–27] The crucial role of SHMT2 in carcinogenesis was also shown for other cancer types, as well as its involvement in clinical cancer drug resistance [28–31].

### SHMT2 activity is inhibited by RNA

**Fig. 2:**
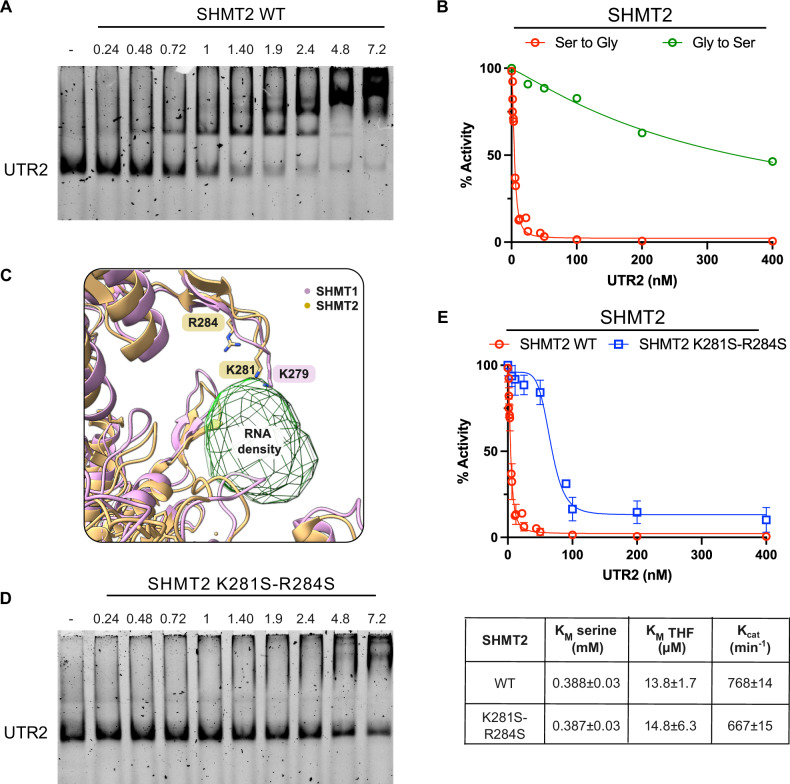
SHMT2 serine cleavage activity is inhibited by RNA. **A** Binding of SHMT2 to UTR2 RNA. EMSA assays with UTR2 RNA and SHMT2 WT. The shift of UTR2 migration indicates that SHMT2 efficiently binds to UTR2 RNA. The apparent Kd of interaction is 0,56 ± 0,05 µM. **B** SHMT2 activity in the presence of UTR2. SHMT2 Ser-to-Gly reaction using L-serine and THF as substrates was carried out in the presence of increasing amount of UTR2 (red symbols) and the corresponding initial velocity, recorded spectrophotometrically, has been plotted as a function of the RNA yielding an IC_50 UTR2_ = 4.4 ± 0.2 nM. Error bars indicate standard deviation (SD) (*n* = 3). Differential effect of UTR2 on the Ser-to-Gly (red) and Gly-to-Ser (green) reaction is also reported. The IC50 of the UTR2 on the serine to glycine reaction is 4.4 ± 0.2 nM, whereas for the glycine to serine conversion the IC50 is 74 times higher (IC50 = 325.4 ± 119,5 nM). Error bars indicate standard deviation (SD) (*n* = 3). **C** Structural superposition of SHMT1:RNA complex (pink) and SHMT2 (beige) structures (PDB: 8A11 [22] and 5V7I [17], respectively). Lysine 279 (SHMT1), 281(SHMT2) and Arginine 284 are highlighted. Lysine 279 (SHMT1) lies on the flap motif and interacts with RNA; by superimposing the SHMT2 structure we identified two positive residues (lysine 281 and arginine 284) on the flap motif that might interact with RNA. **D** EMSA showing SHMT2 K281S-R284S binding to UTR2. The apparent KDapp is 1,6 ± 0,48 µM. **E** Effect of UTR2 on SHMT2 K281S-R284S catalytic activity. Data have been collected following the same protocol reported for the wild-type protein (red line). All the experimental data, acquired in three independent experiments, were fitted to Eq. 2 (see section “Methods”), obtaining the continuous lines shown in the figure. IC50 for the K281S-R284S mutant is 66.4 ± 3.3 nM, 15 times higher than the one observed for SHMT2 WT. The inset shows the KM for serine and THF and the Kcat of SHMT2 WT and mutant obtained experimentally.

Pull-down and RIP-seq assays recently suggested that SHMT2 can control translation of selected mRNAs by interacting with their UTRs regions [32]. We first assessed whether RNA can directly bind to purified SHMT2 with high affinity; the RNA sequence selected for the binding assay was UTR2, well characterized as an interactor and modulator of SHMT1 [21, 22]. Electrophoretic Mobility Shift Assay (EMSA) indicates that SHMT2 binds to UTR2 with an apparent *K*_D_ = 0,56 ± 0,05 µM (Fig. 2A), showing 2.3-fold higher affinity than SHMT1 [21]. The effect of RNA binding on SHMT2 catalysis was assayed by monitoring, in the presence of increasing amount of UTR2, the conversion of saturating L-serine and THF into glycine and 5,10-CH_2-_THF (hereinafter Ser-to-Gly reaction). As shown in Fig. 2B (red curve), addition of UTR2 RNA leads to a dramatic drop in the observed activity, suggesting that UTR2 is a potent inhibitor of Serine cleavage activity (IC_50_ = 4.4 ± 0.2 nM). This inhibition is selective since the glycine to L-serine conversion is only mildly inhibited, as previously observed for the cytosolic SHMT1 [21] (Fig. 2B green curve).

The region involved in RNA binding (Fig. 2C) was predicted by structural superposition of SHMT1:RNA cryoEM complex structure (PDB:8A11 [22], pink) with the SHMT2 crystal structure (PDB:4PVF [33], 5V7I [17], beige), thus identifying Lysine 281 and Arginine 284 as possible contacts stabilizing the RNA ligand in SHMT2 (Fig. 2C). Characterization of the SHMT2 double mutant K281S-R284S confirmed that the positive residues on the conserved flap motif are involved in RNA binding and riboregulation, since the purified mutant protein binds UTR2 with lower affinity and shows a ~ 15 folds higher IC_50_ (Fig. 2D, E).

Taken together, all these data demonstrate that SHTM2 Ser-to-Gly reaction can be efficiently inhibited by UTR2 RNA, with greater efficiency than SHMT1. Given that the serine cleavage reaction, which is the most relevant to cancer metabolism, is mainly carried out in the cells by SHMT2 within the mitochondrial compartment [6, 34], our next goal was to address the inhibitory RNA to this compartment.

### RNA-based mitochondrial SHMT2 targeting

**Fig. 3:**
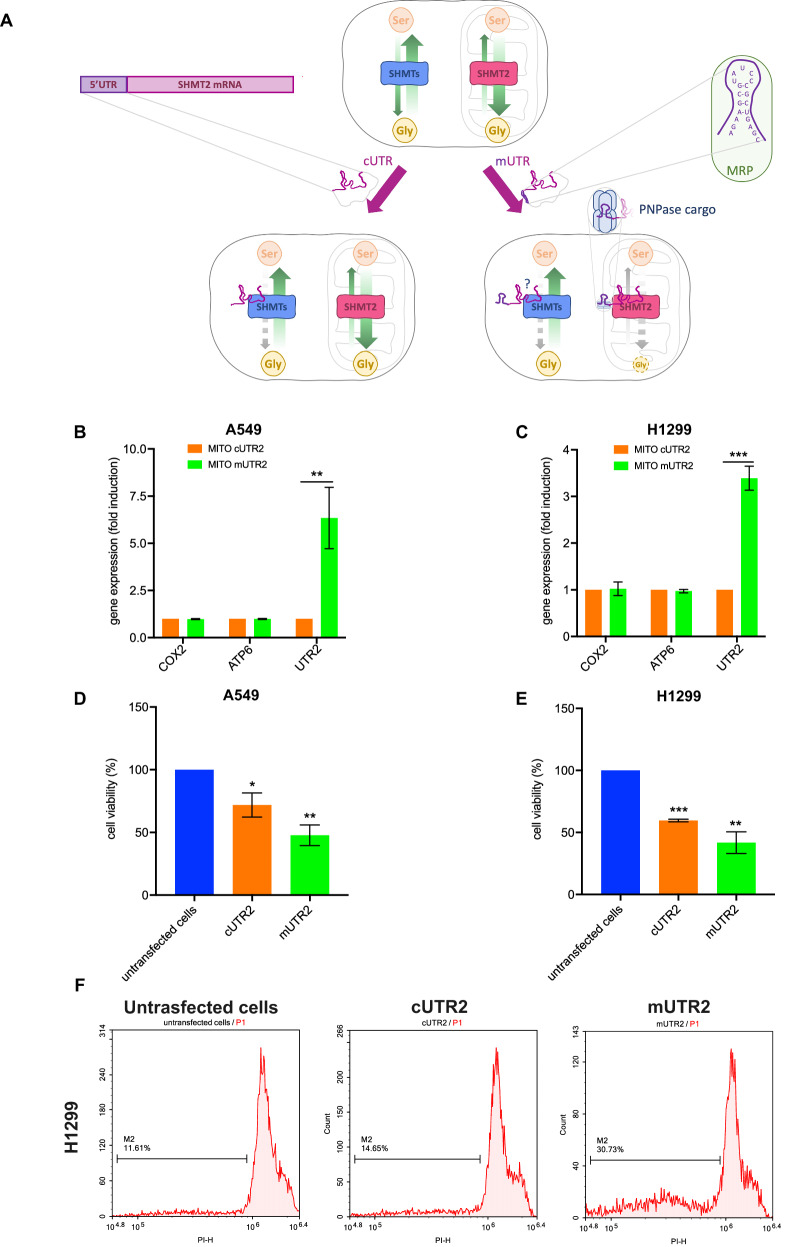
RNA-based mitochondrial SHMT2 targeting. **A** Scheme of the rationale of the experimental design to localize the UTR2 in different compartments. Untreated lung cancer cell is expected to have a steady-state activity of the two SHMT pools sustaining Serine production in the cytoplasm and Glycine production in the mitochondria, carried out by SHMT1/SHMT2 alpha and SHMT2, respectively (upper panel, green arrows in the cartoon). Two different plasmids expressing UTR2 were produced: cUTR2, with only the UTR2 sequence (magenta blow-up in the Figure), and mUTR2, including an additional 5’ sequence for mitochondrial import (violet in the cartoon in the green blow-up). The additional 20 nt sequence derives from the MRP RNA and it is known to be recognized by the PNPase cargo [24], allowing mitochondrial import of RNAs (lower panel, on the right). We expected cUTR2 to mainly target the Ser-to-Gly reaction carried out by the cytoplasmic pool of SHMTs, thus affecting marginally the Ser/Gly steady-state (lower panel, on the left). On the other hand, mUTR2 should mainly target the mitochondrial SHMT2, where the Ser-to-Gly reaction is prevalent, thus in principle affecting more dramatically the Ser/Gly steady-state (lower panel, on the right). An influence of mUTR2 sequence on cytosolic SHMT1 cannot be excluded (question mark in the Figure). Analysis of mitochondrial expression of UTR2 RNAs in A549 and H1299 LUAD cells. qRT-PCR analysis for cUTR2 and mUTR2 performed on mitochondrial extracts from A549 (**B**) and H1299 (**C**) cells transfected with the indicated UTR2 sequences. Data are average ± SD, from three independent experiments. **P < 0.001, ***P < 0.0001. Assessment of cell viability via Trypan Blue exclusion Assay: A549 (**D**) and H1299 (**E**) cells examined 48 h post-transfection with the indicated UTR2 sequences. Data shown as average ± SD from three independent replicates. *P < 0.05, **P < 0.001, ***P < 0.0001. **F** Cell cycle analysis with a focus on the apoptotic population (Sub-G1 Phase) in H1299 examined 48 h post-transfection with the indicated UTR2 sequences Data from a representative of three independent experiments showing a similar trend.

To achieve the goal of mitochondrial targeting, we produced a modified RNA sequence (mUTR2) in which a mitochondrial import signal was located at the 5’ end of the UTR2 RNA. The import signal is based on the MRP RNA tag, which is recognized by polynucleotide phosphorylase (PNPASE), a mitochondrial RNA-import enzyme [24]. For comparison, we also designed a cytoplasmic version of UTR2 (cUTR2) lacking the mitochondrial import tag. The experimental design is summarized in Fig. 3A. To confirm the efficiency of mitochondrial targeting, we transfected A549 and H1299 lung cancer cells with either mUTR2- or cUTR2-expressing vector and performed qRT-PCR on the mitochondrial extracts. The results showed significant enrichment of UTR2 RNA only in the mitochondrial fraction of mUTR2-transfected cells (Fig. 3B, C). These findings validated our strategy for selective mitochondrial RNA delivery. Next, we assessed the functional impact of mUTR2 expression on cell viability. Transfection of mUTR2 resulted in a striking 60% reduction in cell viability, compared to a 30% reduction observed in cUTR2-transfected cells (Fig. 3D, E). These data were further validated on H1299 cells through cell cycle analysis using the Propidium Iodide (PI) assay, which revealed a significant increase in the SubG1 population, indicative of apoptosis, in cells expressing UTR2 sequences. In H1299 cells, apoptosis levels in the cells transfected with the mUTR2 construct were approximately 30%, while lower levels were observed in the cells transfected with cUTR2 (approximately 14%) and control vector (approximately 11%) (Fig. 3F). These results indicate that mitochondrial targeting significantly enhances the inhibitory effects of UTR2 RNA, likely by selectively inhibiting the mitochondrial SHMT2 pool, responsible for the Ser-to-Gly conversion necessary for the survival of cancer cells.

### Specificity of RNA-mediated SHMT2 targeting

**Fig. 4:**
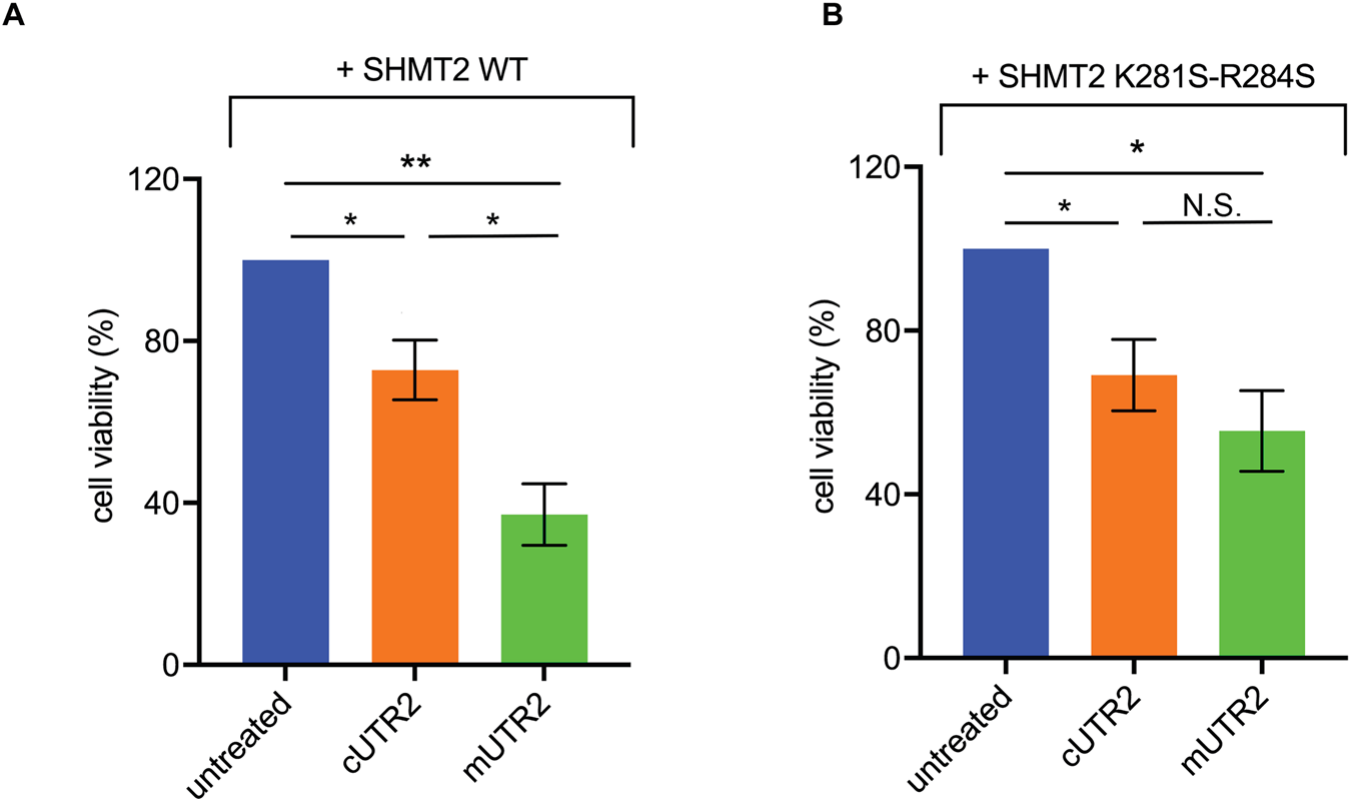
Analysis of SHMT activity and effect of UTR2 in HAP-1 cells. Trypan blue exclusion assay performed on HAP SHMT2KO complemented with SHMT2 WT (**A**) or with SHMT2 K281S-R284S (**B**), after 48 h of transfection with the indicated UTR2 sequences. Data shown as average ± SD from three replicates. n.s.: not significant *P < 0.05, **P < 0.001. Data from individual experiments were normalized to 100%, and subsequent values were calculated relative to this baseline before being averaged.

To confirm that the observed effects of mUTR2 were specifically mediated through its interaction with SHMT2, we generated a cellular model using SHMT2 knockout (SHMT2KO) HAP cells complemented with either wild-type SHMT2 or an RNA-binding-deficient SHMT2 mutant (K281S-R284S). Before evaluating the proper mitochondrial compartmentalization of the proteins, we first assessed the quality of our mitochondrial purification protocol. Western blot analysis (Fig. S1A) demonstrated enrichment of the mitochondrial marker COXIV in the mitochondrial fraction compared to the whole-cell lysate, indicating effective purification. Subsequent Western blot and immunofluorescence analyses confirmed that both wild-type and mutant SHMT2 were successfully expressed and correctly localized to the mitochondria in the complemented cells (Fig. S1B and S2A). We then assessed SHMT enzymatic activity in these cells using a radioisotope-based assay that measures the conversion of serine to glycine. SHMT2KO cells retained approximately 39% of total SHMT activity relative to wild-type HAP cells. Complementation with either wild-type or mutant SHMT2 restored total SHMT activity to levels comparable to those observed in wild-type cells, confirming that both forms of SHMT2 are catalytically active (Fig. S2B). Next, we transfected these HAP1 cell lines with constructs expressing either mUTR2 or cUTR2 and evaluated cell viability. Consistent with previous findings, mUTR2 expression led to a more pronounced reduction in cell viability than cUTR2 in cells expressing wild-type SHMT2 (Fig. 4A). In the SHMT2 K281S-R284S mutant, although the effect of cUTR is comparable to that observed in the recue wt, the reduction of viability observed with the two constructs (i.e. Mutr AND Cutr) is similar (Fig. 4B). These results were further supported by PI staining and analysis of the SubG1 population, which indicated a marked apoptotic response in cells expressing wild-type SHMT2, particularly upon mUTR2 transfection (~23%) compared to cUTR2 (~14%). In contrast, cells expressing the SHMT2 RNA-binding-deficient mutant exhibited only a modest SubG1 increase (~10%), with minimal difference (~2%) between the two constructs, reinforcing the attenuated apoptotic response (Fig. S3). Altogether, these findings demonstrate that the observed reduction in cell viability is critically dependent on the direct interaction between SHMT2 and mUTR2. This supports the specificity of SHMT2 targeting by the RNA-based inhibitor in cancer cells.

### In vivo validation of SHMT2 targeting with UTR2 RNA

**Fig. 5:**
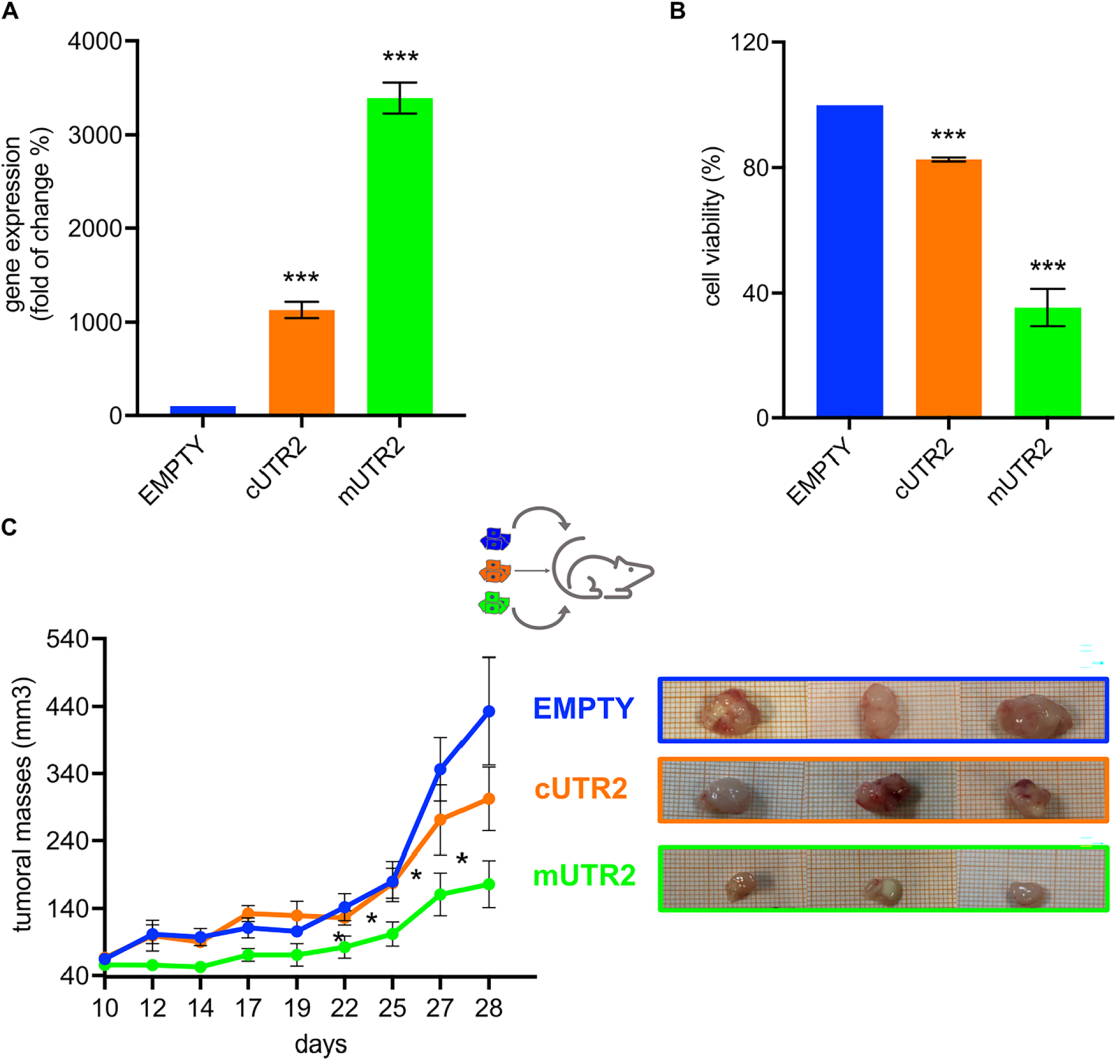
Effect of UTR2 expression on the survival of H1299 cells and in the mouse model. **A** Mitochondrial UTR2 expression: qRT-PCR on mitochondrial extracts of H1299 cells shows higher UTR2 expression in mitochondria when a mitochondrial RNA import signal is employed. Control involved cells transfected with an empty vector, showing low UTR2 expression. Data as average ± SD from three independent replicates. ***P < 0.0001. **B** Trypan blue exclusion assay carried out on H1299 cells lines stably transfected as indicated, 48 h after tetracycline treatment (0.06 µg/µl). Average ± standard deviation (SD) is shown. Data as average ± SD from three independent replicates. ***P < 0.0001. **C** Analysis (left panel) and images (right panel) of tumor volume 30 days after inoculation with the indicated cell lines and treatment with tetracycline in the drinking water. Average ± standard error (SR) is shown. The *p*-values (CTR vs mUTR2) for the last four statistically significant data points are 0.03, 0.05, 0.009, and 0.018, respectively.

To evaluate the therapeutic potential of the delivery of mUTR2 in mitochondria in vivo, we generated H1299 cells stably expressing either the mUTR2 or cUTR2 RNAs under the control of a tetracycline-inducible promoter. An empty vector was used as a control. Tetracycline treatment induced robust expression of UTR2 RNA, as confirmed by qRT-PCR (Fig. S4), with mUTR2 significantly enriched in the mitochondrial fraction of the corresponding stably transfected cell line compared to cUTR2 (Fig. 5A). We first confirmed the impact of mUTR2 expression on cell proliferation in vitro. Tetracycline treatment reduced cell viability by approximately 75% in mUTR2-expressing cells, compared to a 40% reduction in cUTR2-expressing cells (Fig. 5B). Finally, we tested the efficacy of mUTR2 in a xenograft mouse model. Immunocompromised B-NDG mice were subcutaneously injected with H1299 cells stably expressing mUTR2, cUTR2, or an empty vector. Tumor growth was monitored over time following tetracycline administration. Tumor masses in the mUTR2 group were approximately four times smaller than those in the empty vector group and twice smaller than those in the cUTR2 group (Fig. 5C). These results demonstrate that mUTR2-mediated inhibition of SHMT2 significantly suppresses tumor growth in vivo, validating the use of this RNA-based inhibitor to target SHMT2 in the mitochondria in the animal model.

## Discussion

This study represents a major advance in the field of RNA therapeutics, combining riboregulation to target metabolic pathways with the technical achievement of mitochondrial RNA delivery. The idea that RNA molecules can regulate the activity or expression of metabolic enzymes emerged from the discovery of multifunctional enzymes with RNA-binding properties [35]. This finding highlighted the dynamic interplay between RNAs, enzymes, and metabolites, leading to the RNA-enzyme-metabolite (REM) hypothesis. The REM hypothesis bridges cellular metabolism and gene regulation, revealing new layers of metabolic control. Recent research has further supported this concept, demonstrating that RNA molecules can regulate key metabolic enzymes involved in cancer progression. These findings underscore the potential of RNA-based strategies to disrupt tumor metabolism.

Our study represents a significant advancement in the field by presenting the first therapeutic application of RNA-based inhibitors to selectively target the oncogene SHMT2, which currently lacks effective small-molecule inhibitors for in vivo use. SHMT2, a mitochondrial enzyme central to one-carbon metabolism, is often overexpressed in cancer cells [6, 12], where it meets specific metabolic demands and drives tumor progression. Here we demonstrate that the 5′ untranslated region (UTR2) of SHMT2 mRNA can selectively inhibit its activity within the mitochondria. By leveraging this riboregulation mechanism, we provide an innovative strategy to disrupt a metabolic pathway critical for tumor growth. This study marks the first use of riboregulation as an antitumor approach, targeting a metabolic oncogene previously considered “undruggable.”

Additionally, we achieved the first therapeutic delivery of RNA into mitochondria, overcoming one of the major barriers in RNA therapeutics. Mitochondria, with their double-membrane structure and specialized import machinery, have been largely inaccessible to RNA-based therapies. While cytoplasmic and nuclear targeting of RNA therapeutics has advanced, mitochondrial RNA delivery has remained technically challenging. To address this, we designed a mitochondrial-targeting RNA construct (mUTR2) taking advantage of an MRP RNA-derived import signal, which permits RNA transport into mitochondria via PNPASE [24]. Our results demonstrated that mUTR2 efficiently accumulates in mitochondria, successfully overcoming the physical and biochemical barriers of mitochondrial import.

Traditional anticancer drugs, such as methotrexate and 5-fluorouracil (5-FU), have long been the backbone of chemotherapy for OCM-related targets [36, 37]. However, these treatments are often associated with severe side effects, poor specificity, and the rapid emergence of resistance mechanisms [38, 39]. In contrast, our RNA-based therapeutic approach offers a paradigm shift by directly targeting SHMT2 with high specificity, hopefully reducing the likelihood of off-target effects and systemic toxicity. This approach represents a new generation of cancer therapeutics with the potential to outperform current strategies in terms of precision, efficacy, and safety. Further optimization will be necessary to enhance RNA construct(s) stability and therapeutic efficacy. Improvements in RNA modification technologies could extend the half-life of mUTR2 and its derivatives, ensuring sustained therapeutic activity. Additionally, exploring advanced delivery systems such as lipid nanoparticles or exosomes could further improve mitochondrial RNA targeting efficiency and tissue specificity, facilitating clinical translation.

Riboregulation is not limited to SHMT2 but can potentially be applied to control the activity of other RNA-binding metabolic enzymes implicated in cancer and other diseases. The approach holds promise not only for cancer therapy but also for other diseases where mitochondrial dysfunction plays a critical role, such as neurodegenerative diseases, aging, and metabolic disorders [40].

Finally, our work aligns with the broader advancements in RNA-based treatments, including siRNA, antisense RNA, CRISPR guide RNAs (gRNAs), and mRNA vaccines. While these therapies primarily exploit nucleic acid-nucleic acid interactions, our study extends this paradigm by demonstrating the therapeutic potential of nucleic acid-protein interactions via riboregulation. This advancement significantly broadens the scope of RNA-based therapies, offering new possibilities for tackling previously intractable metabolic targets. By bridging the gap between RNA biology and therapeutic innovation, this study paves the way for a new era in precision oncology.

## Material and methods

### Reagents

Chemicals and reagents: unless differently specified and all the salts were obtained from Merck. Tetrahydrofolate and (6S)-5-formyl tetrahydrofolate (monoglutamylated forms) were kindly provided by Merck & Cie (Schaffhausen, Switzerland). All other chemicals were used in the enzymatic assays purchased from Sigma-Aldrich. Antibodies: anti-SHMT2 (sc-390641; Santa Cruz Biotechnology), anti-COXIV (3E11, Cell Signaling) and b-actin (sc-47778; Santa Cruz Biotechnology). Secondary anti-mouse (sc-516102, Santa Cruz Biotechnology), anti-rabbit (sc-2357, Santa Cruz Biotechnology), anti-rabbit FITC (AB_2337977; Jackson ImmunoResearch) and anti-mouse Rhodamine (AB_23387;66 Jackson ImmunoResearch). Kits: QuikChange Lightning Site-Directed Mutagenesis Kit (#210518, Agilent Technologies); the Nucleospin Gel and PCR Purification Mini Kit (W70150-100 Wizbiosolution); Ribomax Large Scale RNA Production System-T7 Kit (P1300, Promega); High-Capacity cDNA Reverse Transcription Kit (4368814, Thermo Fisher Scientific); Sybr SensiFAST Lo-ROX KIT (BIO-94005, Meridian Bioscience); Pierce BCA Protein Assay Kit (23225, Thermo Fisher Scientific).

### Biological resources

Plasmids: pET28b(+) vector was purchased from Novagen (#69744-3); pCMV3 vector was purchased from Sino Biological CV011. Cell lines: A549 and H1299 lung cancer cell lines were purchased from ATCC (Manassas, VA, USA); HAP1 (MALE, Cat#C631; RRID: CVCL_Y019) HAP SHMT1KO and HAP SHMT2KO cell lines were purchased from Horizon Discovery Ltd (Cambridge, UK).

#### Transcriptomic data analysis

Publicly available transcriptomic data for lung adenocarcinoma (LUAD) and normal lung tissues were retrieved from The Cancer Genome Atlas (TCGA) database (https://www.cancer.gov/tcga). A total of 540 LUAD samples and 59 normal lung tissue samples were included in the analysis. Data processing and statistical evaluations were carried out using R (version 4.3.3). Differential expression of SHMT2 between LUAD and normal tissues was assessed using an unpaired two-sample t-test, with a threshold for statistical significance set at *p* < 0.05. Box-and-whisker plots were generated to visualize the distribution of SHMT2 expression across the two groups.

#### Protein expression and purification

Wild-type and mutant (K281S-R284S) SHMT2 genes were cloned into a pET28b vector (Novagen) and expressed as N-terminal histidine-tagged fused protein in *Escherichia coli* (BL21-DE3- C2527H Biolabs). Bacterial cultures were grown at 37 °C in Luria-Bertani (LB) liquid medium supplemented. Purification was performed as previously described by Spizzichino et al. [22]. Briefly, bacterial pellets were then resuspended in a lysis buffer (20 mM Hepes pH 7.2; 250 mM NaCl; 10% glycerol; 40 μM PLP P9255) and lysed by ultrasonic disruption on ice. Soluble proteins were purified by IMAC on a Ni^2+^-His- Trap column (GE Healthcare, Chicago, IL, USA). SHMT2 WT and SHMT2 K281S-R284S eluted with 300 mM imidazole. Fractions containing pure protein were pooled, and imidazole was removed with desalting columns PD10 (GE Healthcare), the histidine tag was then removed by proteolytic digestion with 1U/mg of thrombin (T4648). The digestion mixture was then loaded again on the IMAC on a Ni^2+^-His-Trap column (GE Healthcare, Chicago, IL, USA) and SHMT2 and SHMT2 K281S-R284S, which now bind with low affinity, were eluted with 100 mM imidazole. The proteins were injected into a Superdex 200 column (16/600; GE Healthcare) and eluted as a tetramer with the following buffer, 20 mM Hepes pH 7.2; 250 mM NaCl; 10% glycerol. Protein concentration was determined by measuring the absorbance at 280 nm and applying the Beer-Lambert law (SHMT2 and SHMT2 K281S-R284S ε_280_ 43900 M^−1^cm^−1^). Samples were frozen in liquid nitrogen and stored at −20 °C.

#### Electrophoretic Mobility Shift Assay (EMSA)

EMSA assays were used to assess the binding affinity of different RNAs towards SHMT2, and the ability of the mutants to bind RNA. These experiments were conducted by incubating a fixed amount of RNA with either fixed or increasing concentrations of purified SHMT2 WT or SHMT2 K281S-R284S. All the components were incubated at 25°C for 30 min in 12 μl of binding buffer (20 mM HEPES pH 7.4, 150 mM NaCl) containing 20 μg/ml bovine serum albumin (BSA, 05470), 0,1 ug tRNA (AM7119, Invitrogen), 1 ug/ul RNase inhibitor (N8080119, Invitrogen) and 8% (v/v) glycerol. The reaction mixtures were then subjected to electrophoresis under native conditions using a non-denaturing 4% polyacrylamide gel in 0.5× TBE buffer (45 mM Tris-Borate, 1 mM ethylenediaminetetraacetic acid pH 8.6). For the visualization, gels were stained with SYBR Safe (Invitrogen) in 30 ml of 0.5× TBE and images were acquired using Chemidoc MP Imaging System (Bio-Rad). Densitometric measurements of the free RNA bands were transformed into percentages, the amount of bound RNA (% bound) was then calculated by subtracting the percentage of free RNA from the total, the result was plotted as a function of protein concentration. The apparent dissociation constant (*K*_D_) was estimated fitting the data with the following equation, in which B_max_ corresponds to the maximum binding (100%) and [P] corresponds to the concentration of SHMT2 in the reaction mixture.

1 $$\% {Bound}=\frac{B\max \,\left[P\right]}{{Kd}+\left[P\right]}$$

#### Activity assays

The serine to glycine reaction was measured in 20 mM KPi buffer at pH 7.2 using 0.2 μM enzyme with L-serine and THF as substrates by means of a spectrophotometric coupled assay, in which the 5,10-CH_2_-THF produced by the reaction was oxidized by the NAD-dependent E. coli 5,10-CH_2_-tetrahydrofolate dehydrogenase.

The glycine to serine reaction was measured with 2 μM enzyme in 50 mM Tris HCl at pH 8.8 using a coupled assay in which the L-serine product is oxidized by a NAD^+^-dependent serine dehydrogenase from *Pseudomonas aeruginosa* [41].

The inhibition curves were obtained keeping fixed concentration of substrates (10 mM glycine, 10 mM L-serine, and 50 μM THF, 100 μM 5,10-CH2-THF) while varying the RNA concentration. All obtained inhibition curves were fitted to Eq. 2.

2 $$Y=V\max \left(1-\frac{(\left({x}^{n}\right)}{\left(\left({x}^{n}\right)+K{d}^{n}\right))}\right)+c$$

All spectrophotometric measurements were performed using a Hewlett-Packard 8453 diode-array spectrophotometer. Fitting of data to equations was carried out with the PRISM software (GraphPad, La Jolla, CA, USA).

Measurements of cellular SHMT activity were performed using a radioisotope assay based on the ability of SHMT to catalyze the exchange of the pro-2S proton of glycine with solvent [16]. Cells were detached using trypsin, centrifuged, and washed twice in 2 mL of PBS to remove growth medium. Then, 100 L aliquots of cell suspensions were incubated with [2-3H] glycine (23 mol/L) at 30°C for 3.5 h. Samples were then centrifuged to remove cells and reactions were stopped by the addition of 3% (w/v) trichloroacetic acid to remove radiolabelled glycine. Radioactivity in the solvent was measured as described in [16] and normalized with respect to cells number. The activity of the HAP WT cells was set as 100%.

#### Site-directed mutagenesis

SHMT2 mutants were produced by site-directed mutagenesis using the QuikChange Lightning Site-Directed Mutagenesis Kit (#210518, Agilent Technologies). The pET28b and pCMV3 (Sino Biological) containing the wild-type SHMT2 were used as template DNA. Two complementary oligonucleotides, synthesized by Eurofins, were used as primers for the mutagenesis reactions (primers listed in Table S1). *Escherichia coli* Neb5α cells were transformed to amplify the mutated plasmids and mutagenesis were verified by sequencing. pET28b plasmids containing either SHMT2 WT or SHMT2 K281S-R284S were used for purification of proteins employed to perform all the in vitro experiments. pCMV3 plasmids containing either SHMT2 WT or SHMT2 K281S-R284S were used to obtain the overexpression of the proteins in the *in cellulo* experiments.

#### In vitro transcription

UTR2 sequences was amplified by polymerase chain reaction (PCR) from cDNA of H1299 samples using primers, synthesized by Eurofins, designed to include the T7 promoter sequence upstream of the UTR2 PCR fragment (primers listed in Table S1). PCR products were purified from agarose gel using the Nucleospin Gel and PCR Purification Mini Kit (W70150-100 Wizbiosolution). The DNA fragments generated by PCR were used as templates to produce the corresponding RNA sequences with Ribomax Large Scale RNA Production System-T7 Kit (P1300, Promega).

#### Cell lines

A549 and H1299 lung cancer cell lines were purchased from ATCC (Manassas, VA, USA). The cells were grown in RPMI-1640 medium (R8758, Sigma-Aldrich), supplemented with 100 IU/mL penicillin–streptomycin (P4458; Sigma-Aldrich) and 10% fetal bovine serum (F7524; Sigma-Aldrich). HAP WT, HAP SHMT1KO and HAP SHMT2KO cell lines were purchased from Horizon Discovery Ltd (Cambridge, UK). The cells were grown in IMDM medium (21980-032, Gibco), supplemented with 100 IU/mL penicillin-streptomycin (P4458; Sigma-Aldrich) and 10% foetal bovine serum (F7524; Sigma-Aldrich).

HAP SHMT2KO complemented with SHMT2 WT and K281S-R284S cell lines are obtained after stable transfection of HAP SHMT2KO cells (for details, see transfection session). The cells were grown in IMDM medium (21980-032, Gibco), supplemented with 100 IU/mL penicillin–streptomycin (P4458; Sigma-Aldrich), 10% fetal bovine serum (F7524; Sigma-Aldrich) and 500 µg/ml hygromycin (10687010, Invitrogen).

H1299 tetracycline hygromycin EMPTY, tetracycline hygromycin cUTR2, tetracycline hygromycin mUTR2, are obtained after stable transfection of H1299 cells (for details, see transfection session). The cells were grown in RPMI-1640 medium (R8758, Sigma-Aldrich), supplemented with 100 IU/mL penicillin-streptomycin (P4458; Sigma-Aldrich), 40 µg/ml hygromycin (10687010, Invitrogen) and 10% foetal bovine serum tetracycline free (AU-S181T-500, Aurogene), in this way we were able to control the UTR2 sequence expression that occurred only when we added tetracycline in the culture medium. Cell lines were routinely screened for mycoplasma contamination.

#### RNA extraction and quantitative real-time qRT-PCR analysis

Total RNA was extracted from cells using TRIzol reagent (QG79306, Qiagen) following the manufacturer’s instructions. Reverse transcription reactions were performed on 1 μg of total RNA with High-Capacity cDNA Reverse Transcription Kit (4368814, Thermo Fisher Scientific). Each qPCR tube contained 5 μl Sybr SensiFAST Lo-ROX KIT (BIO-94005, Meridian Bioscience), 0.4 μl primer forward 10 µM, 0.4 μl primer reverse 10 µM, 1 μl complementary DNA (1:10 diluted) and 3.2 μl nuclease-free water (129115, Qiagen), to achieve the final volume 10 μl. Reactions were performed using QuantStudio 3 by Applied Biosystems (Thermo Fisher Scientific). The cycling protocol was 95 °C for 2 min, 40 cycles of 95 °C for 5 s, 60 °C for 30 s, before fluorescence detection. A melting curve was determined using the standard instrument protocol. Amplification specificity was assessed by analysis of melting curves. Relative fold change of the expression of individual genes was calculated using the 2 − ΔΔCt. ΔCt is calculated as the difference between Ct of each gene and Ct of β-actin gene, used as a normalizer. Each sample was analyzed in triplicate. Primer sequences are shown in Table S1.

#### Protein extraction and Western blotting analysis

Cells were harvested and lysed with CelLytic (C2978, Sigma-Aldrich) and protein concentration was determined using the Pierce BCA Protein Assay Kit (23225, Thermo Fisher Scientific). 20 µg of proteins were separated by 4–15% gradient SDS-PAGE electrophoresis. Briefly, the proteins were then transferred onto nitrocellulose membranes and incubated overnight at 4 °C with the primary antibody: anti-SHMT2 (dilution 1:1000, sc-390641; Santa Cruz Biotechnology), anti-COXIV (dilution 1:1000, 3E11, Cell Signaling) and b-actin (dilution 1:5000, sc-47778; Santa Cruz Biotechnology). The day after were incubated with the respective horseradish peroxidase-conjugated secondary anti-mouse (1:5000, sc-516102, Santa Cruz Biotechnology) and anti-rabbit (1:5000, sc-2357, Santa Cruz Biotechnology) antibody for 1 h at RT. Membranes were washed with PBS- 0.1% Tween 20 and developed using the chemiluminescence system Chemidoc MP Imaging System (Bio-Rad).

#### DNA cells transfection

1 × 10^5^cells/ml A549, H1299, HAP-SHMT2KO complemented with SHMT2 WT and HAP-SHMT2KO complemented with SHMT2 K281S-R284S cells were seeded and transiently transfected with 1 µg of cUTR2 and/or mUTR2 sequences from GenScript using JetPrime (Polyplus transfection) according to the manufacturer’s instructions. Structures of the plasmids are shown in Table S2. The cells were used for trypan blue exclusion assay 48 h after transfection, or 24 h after transfection for mitochondria purification. H1299 and HAP-SHMT2KO were stable transfected respectively with “tetracycline hygromycin UTR2 sequences” plasmids and with SHMT2 WT/SHMT2 K281S-R284S plasmids using JetPrime (Polyplus transfection) according to the manufacturer’s instructions. Structures of the plasmids are shown in Table S2. 48 h after transfection hygromycin was added to the culture medium (500 µg/ml for HAP cells, 40 µg/ml for H1299): the selection process went on for 2–3 weeks until we isolated only stable transfected cell clones. After selection, the cells were always grown in the respective medium containing hygromycin, at the same concentration used during the selection process.

#### Mitochondria purification

Mitochondria were extracted using the Mitochondria Isolation Kit for Mammalian Cells (89874, Thermo Scientific) according to the manufacturer’s instructions. The mitochondrial extracts were than used for RNA and protein extraction.

#### Trypan blue exclusion assay

The growth medium was collected, the cells were washed once with phosphate-buffered saline (PBS). Adherent cells were removed by treatment with trypsin, which in turn was blocked using complete medium. All the collected cell fractions were centrifuged 5 min at 1000 rpm and the supernatant carefully discarded. The harvested cells were then washed with PBS, centrifuged 5 min at 1000 rpm and the supernatant discarded. Following the addition of a 0.4% (w/v) trypan blue stain solution (EBT-001, NanoEntek) cells were transferred to a cell counting slide (EVS-050, EveTM NanoEntek), visualized, and counted using EveTM Automatic Cell Counter, (NanoEntek). Cells stained with trypan blue dye were considered nonviable.

#### Cell cycle analysis

Cells were harvested, washed twice in PBS, and fixed dropwise in 70% cold ethanol. After fixation, cells were washed with PBS and incubated for 3 h at room temperature in the dark with a staining solution containing 10 μg/mL propidium iodide (Sigma-Aldrich) in PBS. Flow cytometric analysis was performed using a NovoCyte 1040 (Agilent Technologies) equipped with a 488 nm laser. Doublets were excluded, and cell cycle phases were evaluated based on DNA content (G0/G1, S, G2/M). Sub-G1 events were considered as apoptotic nuclei.

#### Immunofluorescence

HAP cells were grown on coverslips and fixed in 3.7% paraformaldehyde—30 mM sucrose for 10 min. Afterwards, cells were permeabilized in 0.1% Triton X-100 and blocked in 3% bovine serum albumin in PBS- 0.05% Tween-20 for 1 h at room temperature. The incubation with primary antibodies was performed at 4°C overnight in a dark humidity chamber. Subsequently, the fluorescence-labeled secondary antibodies were added in PBS containing 0.05% Tween 20 and 3% BSA solution and incubated at room temperature for 30 min. Cells were counterstained with DAPI (0.1 μg/ml) and mounted using the mounting media. Primary antibodies were anti-SHMT2 (dilution 1:50, sc-390641; Santa Cruz Biotechnology) and anti-COXIV (dilution 1:100, 3E11, Cell Signaling). Secondary antibodies were anti-rabbit FITC (dilution 1:50, AB_2337977; Jackson ImmunoResearch) and anti-mouse Rhodamine (dilution 1:50, AB_23387;66 Jackson ImmunoResearch). Samples were acquired using an Eclipse 90i microscope (Nikon Instruments S.p.A., Campi Bisenzio Firenze, IT, USA) equipped with 40× (N.A. 0.75) and 100× (oil immersion, N.A. 1.3) objectives and a Qicam Fast 1394 CCD camera (QImaging Surrey, BC, Canada). Images were acquired using NIS-ELEMENTS AR 3.2 (Nikon Instruments S.p.A.); elaboration and processing was performed using NIS-ELEMENTS HC 5.02 (Nikon Instruments S.p.A.) and GIMP - GNU IMAGE MANIPULATION PROGRAM.

#### Xenograft tumor assay

All animal experiments were carried out under the authorization n° 312/2021-PR of the Italian ministry of Health. In vivo experiments were conducted at PLAISANT COMPANY (Rome, Italy), a specialized and licensed facility for live animal experimentation. Male mice aged 6–8 weeks strand NOD.CB17-Prkdcscid IL2rgtm1/BcgenHsd (B-NDG) were purchased from ENVIGO company. Mice were randomly assigned to three groups, eight mice per group: one groups for injection with H1299 TET HYGRO EMPTY, one groups for H1299 TET HYGRO cUTR2, one groups for H1299 TET HYGRO mUTR2. Each mouse was injected in the flank with the respective cell line (1 × 10^7^ cells for mouse). The tumor mass started to be formed a week after injection. In the drinking water there was 150 µg/ml tetracycline and 1% sucrose, the water is changed every 2–3 days in a week. Mice were sacrificed after 30 days. Tumor dimensions were measured using calipers and the tumor masses *w* were calculated at each time *t* with the following formula:

$$w=\frac{l* z}{2}\rho$$

where *l* is the length of the tumor measured in mm and *z* is the tumor squared width expressed in mm^2^.

The maximal tumor size/burden permitted by the Italian Ministry of Health is of 800–1000 mm^3^. The biggest tumor size detected in our experiments was of 730,83 mm^3^.

### Mice model

Male mice aged 6–8 weeks.(NOD.CB17-Prkdcscid IL2rgtm1/BcgenHsd). At the time of the experiment, mice had body weights of 26.2 ± 0.5 g (mean ± SEM).

#### Statistical considerations

Unless otherwise specified, statistical significance was assessed using ANOVA followed by Bonferroni post hoc test for direct comparisons.

#### Statistical power in the in vivo study

In this experiment, we expect a mean difference (Δ\DeltaΔ) of 200 mm³ between two independent groups. The worst-case scenario assumes a standard deviation (SD) of approximately 130 mm³. The statistical analysis uses t-test or ANOVA + Bonferroni correction, purpose: Compare the means of two groups to determine if the observed difference is statistically significant.

Key input parameters

- Effect Size (Cohen’s d): 1.538
- Significance Level (α): 0.05 Statistical Power (1-β): 0.8
- Allocation Ratio (N2/N1): 1

Results

- Noncentrality parameter (δ): 3.077
- Critical t-value: 2.145
- Degrees of Freedom (Df): 14
- Sample Size per Group: 8 participants per group.
- Actual Power: 0.816

#### Statistical analysis in the in vivo studies

All the data are the mean ± standard deviation of at least three independent biological experiments, each repeated in three technical replicates. All the statistical analyses were performed using one-way ANOVA followed by the Bonferroni post-hoc comparison test. A *p* < 0.05 was considered significant where the exact p-value is not reported. *p < 0.05; **p < 0.001; ***p < 0.0001.

## Supplementary information

Figure S1. Analysis of SHMT2 expression in HAP cell line

Figure S2. Immunofluorescence analysis for SHMT2 localization in HAP cell line and cellular activity assay

Figure S3. Evaluation of the Sub-G1 population in HAP Cells Transfected with Plasmids Expressing cUTR or mtUTR2.

Figure S4. UTR2 expression evaluated by qRT-PCR in H1299 cells transfected with mUTR2 under a Tetracycline-inducible system.

Table S1. Nucleotide sequences of the primers used in the studies (written 5' to 3').

Table S2. Structure of the plasmids used in the studies.

uncropped western blot

## Data Availability

All data supporting this study are presented in this published article and in its Supplementary information files. Full and uncropped western blots are available as supplemental material.
