# Supplementary material for: RNA-mediated inhibition of mitochondrial SHMT2 impairs cancer cell proliferation
Source: Cell Death Discov. 2025 Aug 6;11:369. doi: 10.1038/s41420-025-02646-y (PMC12328718; doi:10.1038/s41420-025-02646-y)
Supplement: Supplementary file 1 — Figure S1. Analysis of SHMT2 expression in HAP cell line [file 41420_2025_2646_MOESM1_ESM.pdf]

A

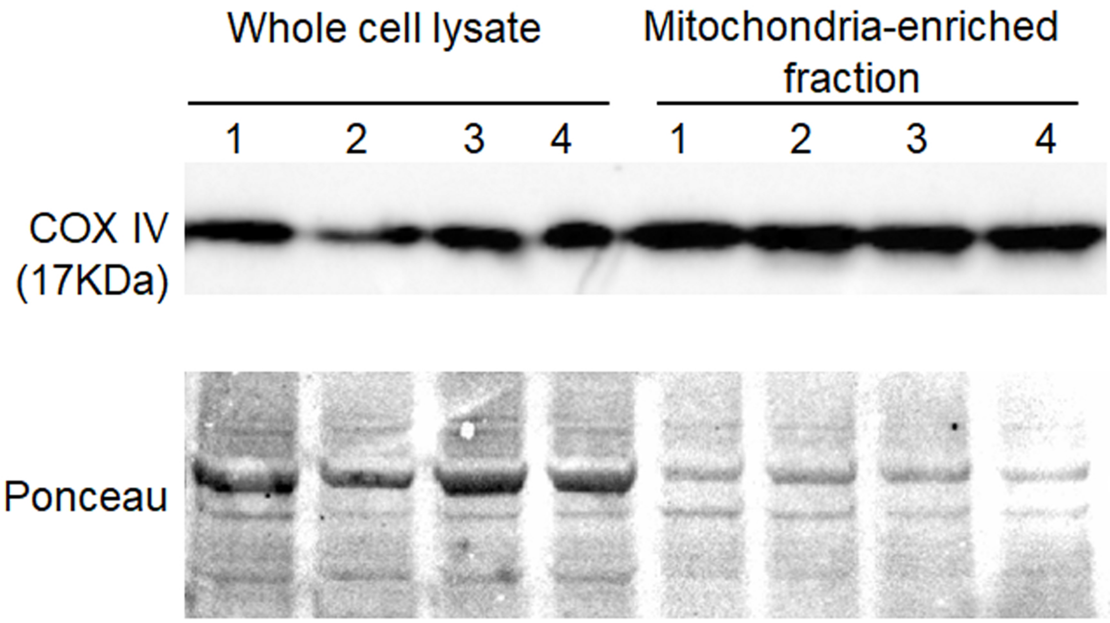

1=HAP WT
2=HAP SHMT2KO
3=HAP SHMT2KO + SHMT2 WT
4=HAP SHMT2KO + SHMT2 K281S-R284S

B

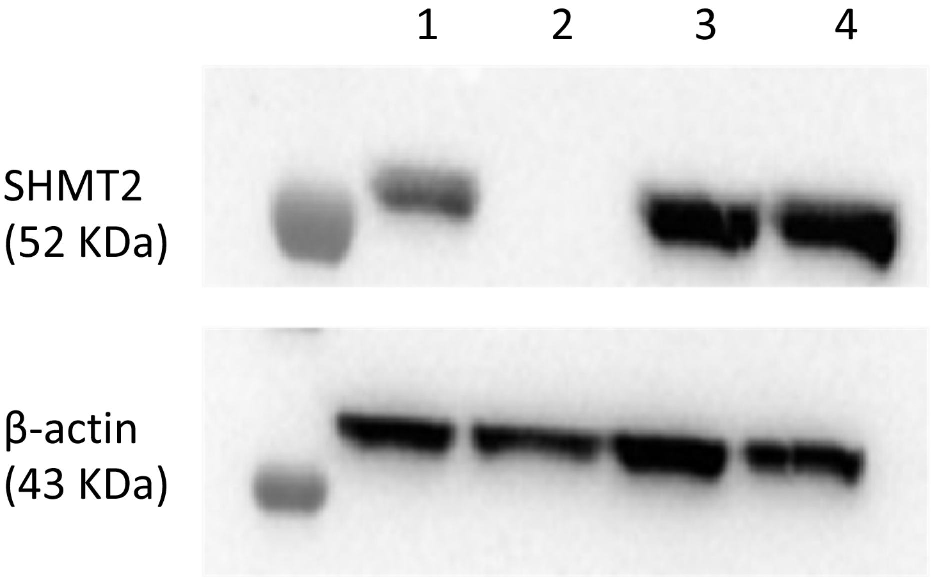

1=HAP WT
2=HAP SHMT2KO
3=HAP SHMT2KO + SHMT2 WT
4=HAP SHMT2KO + SHMT2 K281S-R284S

Figure S1. Analysis of SHMT2 expression in HAP cell line. A) Western Blot of total and mitochondrial extracts and B) Western Blot of mitochondrial extracts of HAP WT, HAP SHMT2KO, and HAP SHMT2KO complemented with SHMT2 WT or SHMT2 K281S-R284S are shown. Antibody details in materials and methods.
