# Supplementary material for: RNA-mediated inhibition of mitochondrial SHMT2 impairs cancer cell proliferation
Source: Cell Death Discov. 2025 Aug 6;11:369. doi: 10.1038/s41420-025-02646-y (PMC12328718; doi:10.1038/s41420-025-02646-y)
Supplement: Supplementary file 2 — Figure S2. Immunofluorescence analysis for SHMT2 localization in HAP cell line and cellular activity assay [file 41420_2025_2646_MOESM2_ESM.pdf]

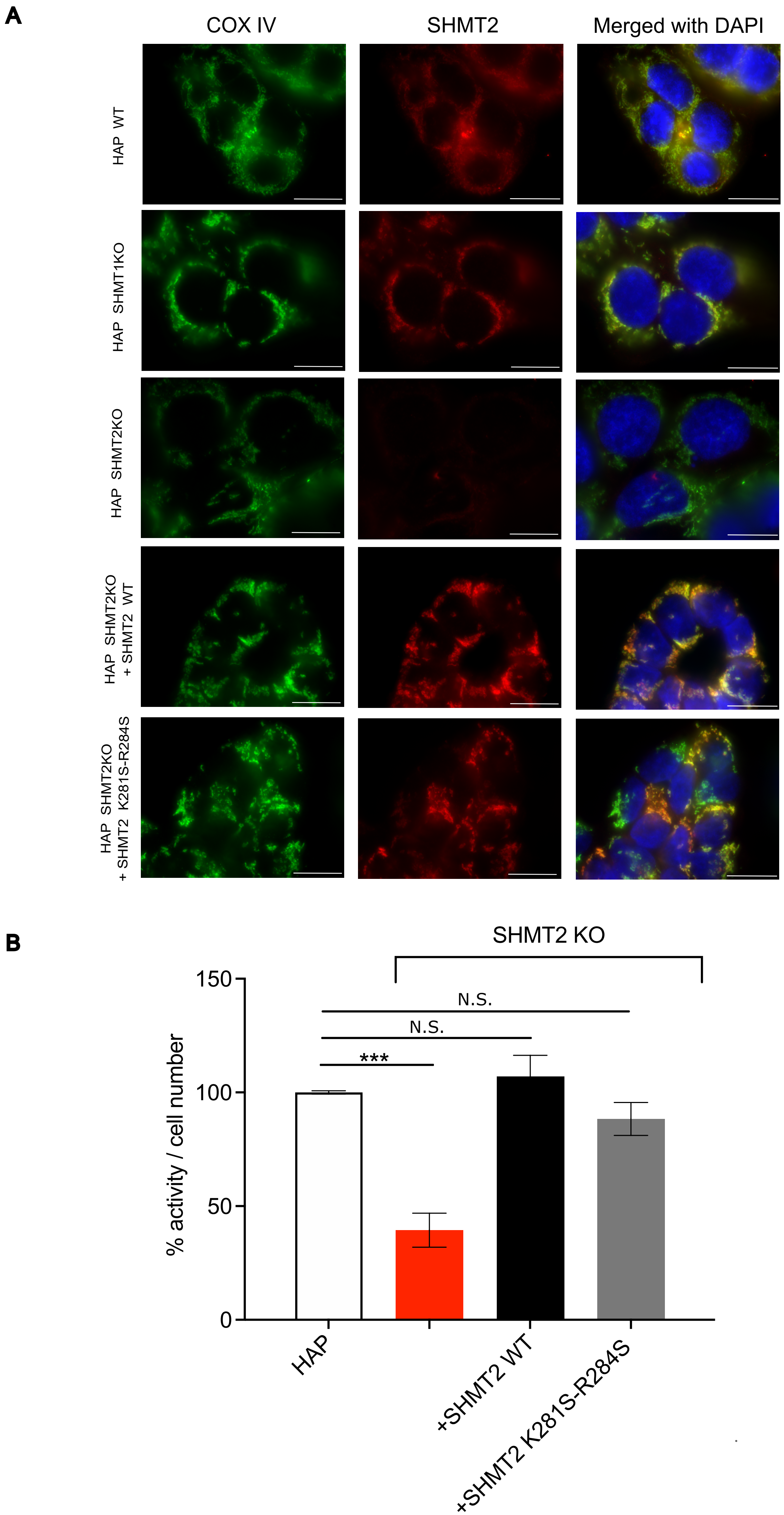
A
COX IV
SHMT2
Merged with DAPI
HAP WT
HAP SHMT1KO
HAP SHMT2KO
HAP SHMT2KO + SHMT2 WT
HAP SHMT2KO + SHMT2 K281S-R284S
B
SHMT2 KO
N.S.
N.S.
***
% activity / cell number
150
100
50
0
HAP
+SHMT2 WT
+SHMT2 K281S-R284S

Figure S2*. *Immunofluorescence analysis for SHMT2 localization in HAP cell line and cellular activity assay. **A)** The localization of SHMT2 WT and mutant on HAP wild-type (WT), SHMT2 knock-out (SHMT2KO), SHMT2KO complemented with SHMT2 WT, and SHMT2KO with K281S-R284S mutant is shown. Scale bar: 10 µm. Antibody details in materials and methods. **B)** Total SHMT enzymatic activity measured by tritium-exchange radioisotopic assay on HAP, HAP SHMT2 KO, HAP SHMT2 KO cells complemented with plasmids encoding WT or K281S-R284S SHMT2. The graphs represent three independent experimental replicates. Data shown as average ± SD from three replicates *** P<0.0001
