# Supplementary material for: RNA-mediated inhibition of mitochondrial SHMT2 impairs cancer cell proliferation
Source: Cell Death Discov. 2025 Aug 6;11:369. doi: 10.1038/s41420-025-02646-y (PMC12328718; doi:10.1038/s41420-025-02646-y)
Supplement: Supplementary file 3 — Figure S3. Evaluation of the Sub-G1 population in HAP Cells Transfected with Plasmids Expressing cUTR or mtUTR2. [file 41420_2025_2646_MOESM3_ESM.pdf]

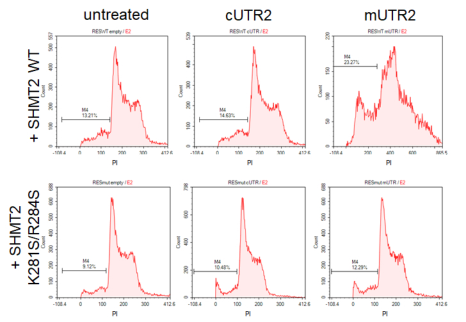

untreated
cUTR2
mUTR2
+ SHMT2 WT
+ SHMT2 K281S/R284S
RESWT empty / E2
RESWT cUTR / E2
RESWT mUTR / E2
RESmut empty / E2
RESmut cUTR / E2
RESmut mUTR / E2
M4 13.21%
M4 14.63%
M4 23.27%
M4 9.12%
M4 10.48%
M4 12.29%
Count
PI

Figure S3. Evaluation of the Sub-G1 population in HAP Cells Transfected with Plasmids Expressing cUTR or mtUTR2. HAP SHMT2KO Cells Were Complemented with Either SHMT2 WT or the SHMT2 K281S-R284S Mutant and Analyzed 48 Hours After Transfection with the Indicated UTR2 Constructs. Data shown are from one representative experiment out of three independent replicates that yielded similar results.
