# Supplementary material for: RNA-mediated inhibition of mitochondrial SHMT2 impairs cancer cell proliferation
Source: Cell Death Discov. 2025 Aug 6;11:369. doi: 10.1038/s41420-025-02646-y (PMC12328718; doi:10.1038/s41420-025-02646-y)
Supplement: Supplementary file 4 — Figure S4. UTR2 expression evaluated by qRT-PCR in H1299 cells transfected with mUTR2 under a Tetracycline-inducible system. [file 41420_2025_2646_MOESM4_ESM.pdf]

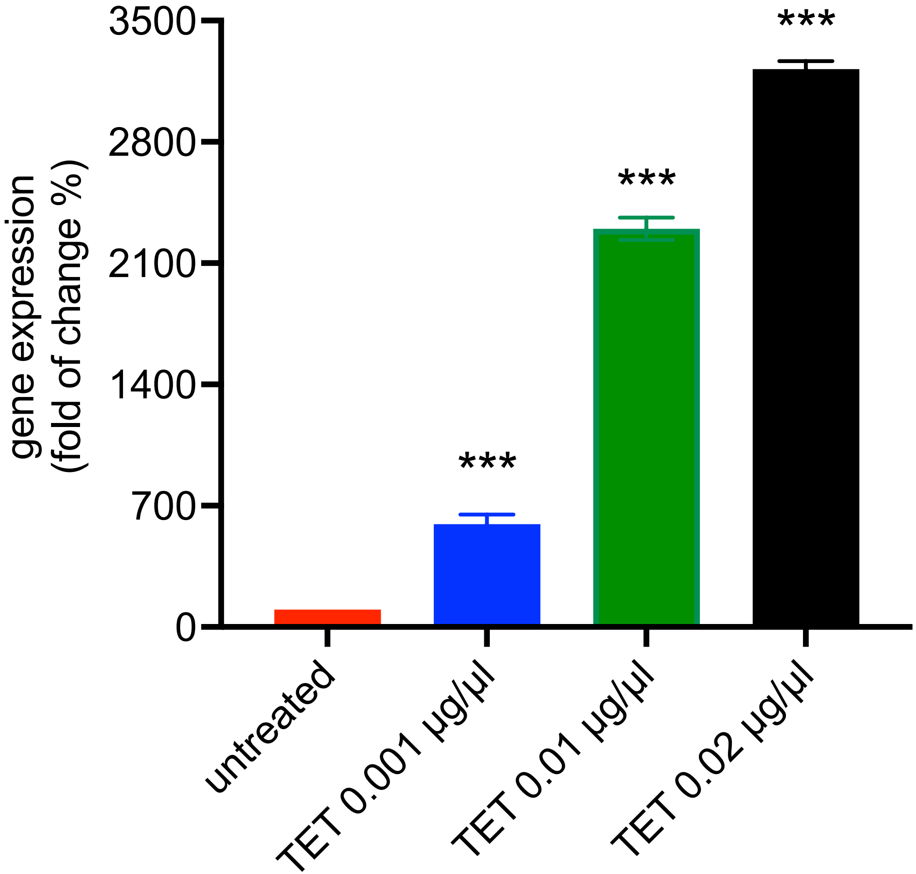
3500
2800
2100
1400
700
0
gene expression
(fold of change %)
***
***
***
untreated
TET 0.001 µg/µl
TET 0.01 µg/µl
TET 0.02 µg/µl

Figure S4. UTR2 expression evaluated by qRT-PCR in H1299 cells transfected with mUTR2 under a Tetracycline-inducible system. Pre-transfection treatment involved varying tetracycline concentrations. Post-48h transfection, RNA was extracted for analysis. Data shown as average ± SD from three independent experiments.*** $P<0.0001$.
