# Supplementary material for: RNA-mediated inhibition of mitochondrial SHMT2 impairs cancer cell proliferation
Source: Cell Death Discov. 2025 Aug 6;11:369. doi: 10.1038/s41420-025-02646-y (PMC12328718; doi:10.1038/s41420-025-02646-y)
Supplement: Supplementary file 5 — Table S1. Nucleotide sequences of the primers used in the studies (written 5' to 3'). [file 41420_2025_2646_MOESM5_ESM.pdf]

	In vitro **Transcription**
UTR2	Fw: TAATACGACTCACTATAGGGATAAAGAAAAAAGCGGTGAG<br>Rv: CGCAACTCGGAAGTCGCAGGAG
	**Mutant Site-Directed Mutagenesis**
SHMT2 K281S-R284S	Fw: GAAGGCTGTGGACCCCAGCACTGGCAGCGAGATCCCTTACACATTTG<br>Rv: CAAATGTGTAAGGGATCTCGCTGCCAGTGCTGGGGTCCACAGCCTTC
	**Real-Time PCR**
UTR2	Fw: GGCGAACTACAATTCCCAAA<br>Rv: ACTCTGGTCTAGGGCAGCAA
β-ACTIN	Fw: AATGTGGCCGAGGACTTTGAT<br>Rv: AGGATGGCAAGGGACTTCCTG
COX2	Fw: ACCGTCTGAACTATCCTGCC<br>Rv: AGATTAGTCCGCCGTAGTCG
ATP6	Fw: GCCACCTACTCATGCACCTA<br>Rv: CGTGCAGGTAGAGGCTTACT

Table S1. Nucleotide sequences of the primers used in the studies (written 5' to 3'). Forward primers used to amplify the constructs employed in the *in vitro* transcription experiments contain the T7 promoter sequence at the 5' terminal (underlined in the text).
