# Supplementary material for: RNA-mediated inhibition of mitochondrial SHMT2 impairs cancer cell proliferation
Source: Cell Death Discov. 2025 Aug 6;11:369. doi: 10.1038/s41420-025-02646-y (PMC12328718; doi:10.1038/s41420-025-02646-y)
Supplement: Supplementary file 6 — Table S2. Structure of the plasmids used in the studies. [file 41420_2025_2646_MOESM6_ESM.pdf]

<table>
<tr><td></td><td>Vectors</td></tr>
<tr><td>Structure of plasmids containing the cUTR2 and mUTR2 sequences.

In both cases <u>CMV promoter</u> drives the expression of the sequences.</td><td>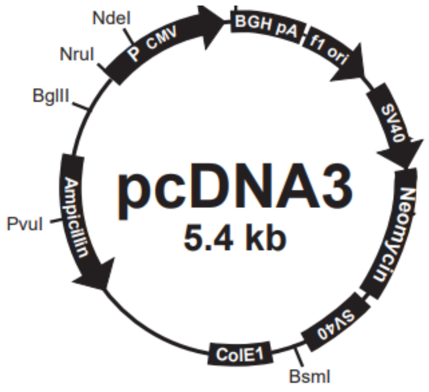
</td></tr>
<tr><td>Structure of plasmids containing the SHMT2 WT and SHMT2 K281S-R284S, used for in vitro experiments.

In both cases <u>T7 promoter</u> drives the expression of the sequences.</td><td>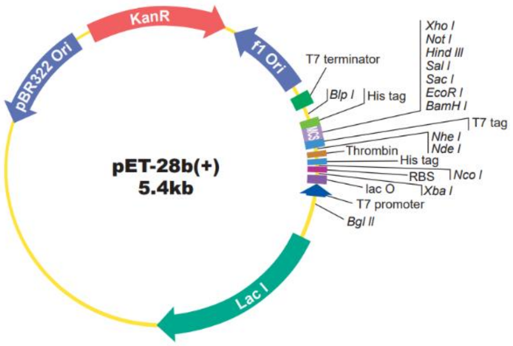
</td></tr>
<tr><td>Structure of plasmids containing the SHMT2 WT and SHMT2 K281S-R284S, used for in cellulo experiments.

In both cases <u>CMV promoter</u> drives the expression of the sequences.</td><td>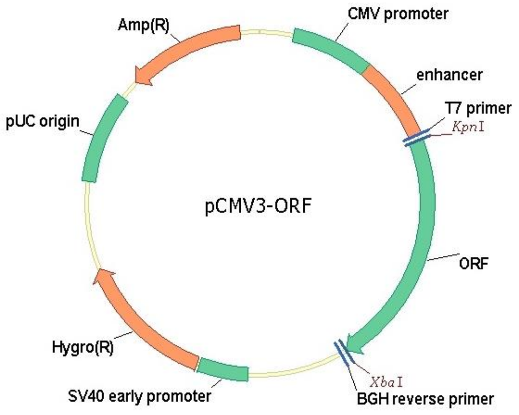
</td></tr>
</table>

Table S2. Structure of the plasmids used in the studies.
