# Supplementary material for: RNA-mediated inhibition of mitochondrial SHMT2 impairs cancer cell proliferation
Source: Cell Death Discov. 2025 Aug 6;11:369. doi: 10.1038/s41420-025-02646-y (PMC12328718; doi:10.1038/s41420-025-02646-y)

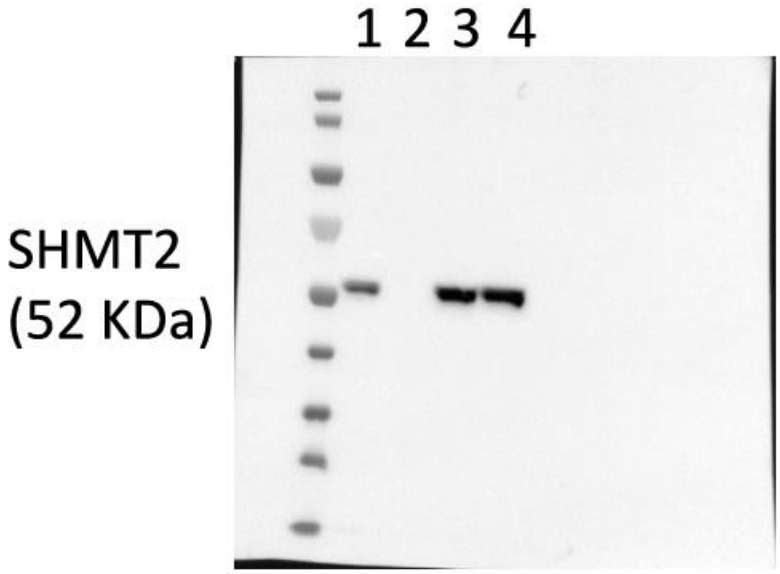

1=HAP WT
2=HAP SHMT2KO
3=HAP SHMT2KO + SHMT2 WT
4=HAP SHMT2KO + SHMT2 K281S-R284S

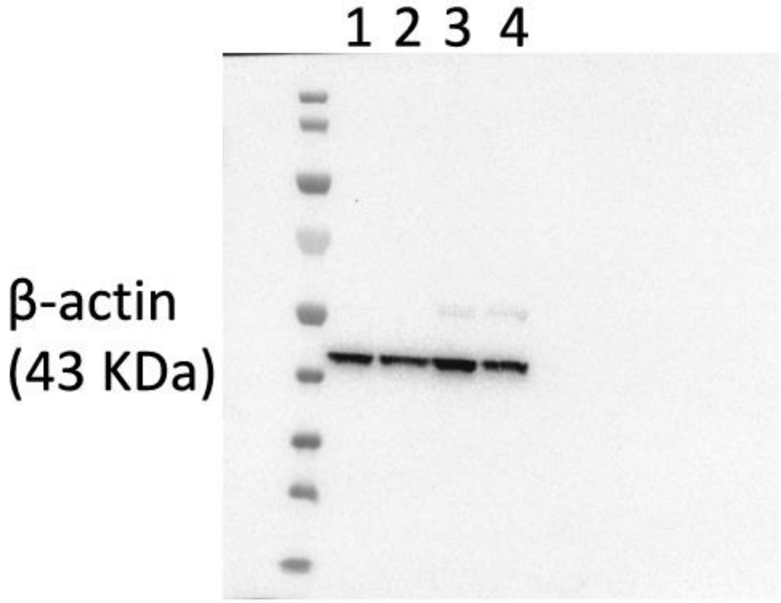

1=HAP WT
2=HAP SHMT2KO
3=HAP SHMT2KO + SHMT2 WT
4=HAP SHMT2KO + SHMT2 K281S-R284S

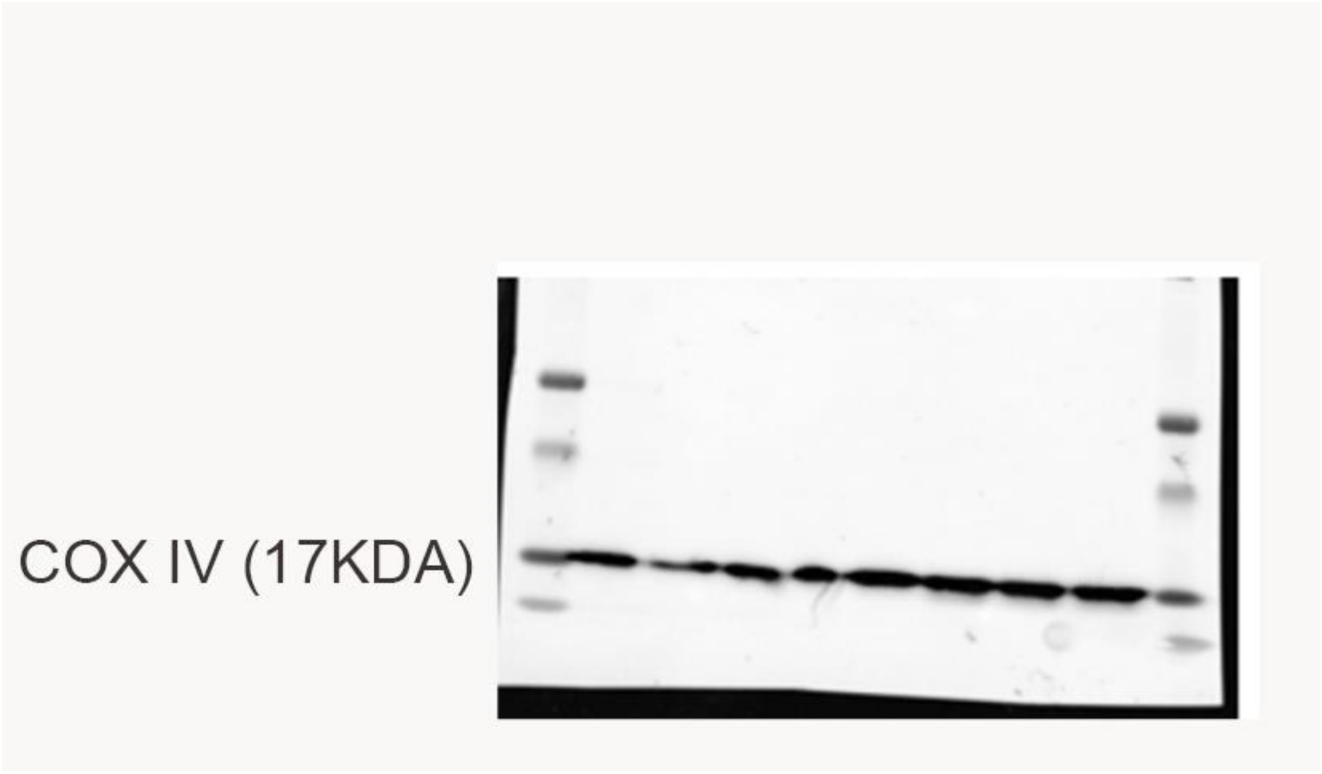

Supplement: Supplementary file 7 — uncropped western blot [file 41420_2025_2646_MOESM7_ESM.pdf]
